# Claudin 7 expression and localization in the normal murine mammary gland and murine mammary tumors

**DOI:** 10.1186/bcr988

**Published:** 2005-01-17

**Authors:** Brigitte Blackman, Tanya Russell, Steven K Nordeen, Daniel Medina, Margaret C Neville

**Affiliations:** 1 University of Colorado Health Sciences Center, Denver, Colorado, USA; 2 Baylor College of Medicine, Houston, Texas, USA

**Keywords:** claudin, EPH4 cells, mammary development, mammary tumors, tight junction

## Abstract

**Introduction:**

Claudins, membrane-associated tetraspanin proteins, are normally associated with the tight junctions of epithelial cells where they confer a variety of permeability properties to the transepithelial barrier. One member of this family, claudin 7, has been shown to be expressed in the human mammary epithelium and some breast tumors. To set the stage for functional experiments on this molecule, we examined the developmental expression and localization of claudin 7 in the murine mammary epithelium and in a selection of murine mammary tumors.

**Method:**

We used real-time polymerase chain reaction, *in situ *mRNA localization, and immunohistochemistry (IHC) to examine the expression and localization of claudin 7. Frozen sections were examined by digital confocal microscopy for colocalization with the tight-junction protein ZO1.

**Results:**

Claudin 7 was expressed constitutively in the mammary epithelium at all developmental stages, and the ratio of its mRNA to that of keratin 19 was nearly constant through development. By IHC, claudin 7 was located in the basolateral part of the cell where it seemed to be localized to discrete vesicles. Scant colocalization with the tight-junction scaffolding protein ZO1 was observed. Similar results were obtained from IHC of the airway epithelium and some renal tubules; however, claudin 7 did partly colocalize with ZO1 in EPH4 cells, a normal murine mammary cell line, and in the epididymis. The molecule was localized in the cytoplasm of MMTV-*neu *and the transplantable murine tumor cell lines TM4, TM10, and TM40A, in which its ratio to cytokeratin was higher than in the normal mammary epithelium.

**Conclusion:**

Claudin 7 is expressed constitutively in the mammary epithelium at approximately equal levels throughout development as well as in the murine tumors examined. Although it is capable of localizing to tight junctions, in the epithelia of mammary gland, airway, and kidney it is mostly or entirely confined to punctate cytoplasmic structures, often near the basolateral surfaces of the cells and possibly associated with basolateral membranes. These observations suggest that claudin 7 might be involved in vesicle trafficking to the basolateral membrane, possibly stabilizing cytoplasmic vesicles or participating in cell–matrix interactions.

## Introduction

The claudins comprise a large family of tetraspanin membrane proteins thought to be the major barrier-forming proteins of tight junctions, the cell–cell contacts at the apical border of epithelial cells that control the paracellular movement of solutes. These proteins are highly conserved, with four transmembrane domains and two hydrophobic extracellular loops; the latter are thought to mediate cell–cell adhesion [1] and to confer specific paracellular permeability properties on cell monolayers [2,3]. Claudin 7 shares the general structural characteristics of the family, differing primarily in its amino-terminal cytoplasmic tail [4]. The molecule has been shown to be associated with epithelial cells in the human breast [5], and its loss is associated with some breast and head and neck malignancies [5,6]. It has been shown to be expressed in parts of the renal tubule [7] and the airway epithelium [8], where it is localized to the basolateral aspects of the cells. Here we show that claudin 7 is constitutively present in the epithelium of the murine mammary gland, again localized, not to tight junctions but to punctate structures at or near the basolateral surfaces of the cells. It was present at all cell borders of several murine mammary tumors. Nonetheless, the protein can localize to tight junctions, as shown by its partial colocalization with ZO1 in cultured mammary epithelial cells and epididymis, suggesting a possible dual function depending on tissue type.

## Method

### Animals and tissue preparation

CD-1 mice, purchased from Charles River Breeding Laboratory (Wilmington, DE), were maintained in the USDA-approved Animal Resource Center of the University of Colorado Health Sciences Center. All procedures were approved by the Institutional Animal Care and Use Committee. The fourth mammary glands of virgin female mice at 3, 6, and 12 weeks of age, of female mice during early gestation (5–7 days), mid-gestation (12 days) and late gestation (18 days), at days 2 and 10 of lactation, and at days 21 and 29 of involution were collected after killing with a lethal dose of pentobarbital. Liver, lung, and kidneys were obtained from virgin female mice and epididymis from male mice. The day on which vaginal plugs were observed was counted as day one of pregnancy. Mice overexpressing the *Her2*/*neu *oncogene [9] were obtained from Jackson Laboratories, and mammary tumors were dissected and frozen when they reached about 0.5 cm in diameter. The transplantation tumor models TM4, TM10L, and TM40 are maintained in the Medina laboratory as described [10]. Tissues were flash-frozen in liquid nitrogen in Tri-Zol reagent (Gibco BRL, Life Technologies) for total RNA purification. For protein extractions, the tissue was washed twice in 1 ml of phosphate-buffered saline (PBS) and then placed in 3 volumes of 1% Nonidet P40 buffer (25 mM Hepes-NaOH, pH 7.4; 150 mM NaCl, 4 mM EDTA, 1% Nonidet P40) containing protease inhibitors (10 μg/μl leupeptin, 10 μg/μl aprotinin, 10 μM pepstatin A, 1 mM phenylmethylsulphonyl fluoride, in dimethyl sulphoxide). For hybridizations and immunohistochemistry of frozen tissue *in situ*, tissues were placed in Tissue-Tek OCT 4583 Compound (Sakura, Torrance, CA) and flash-frozen in isopentane and liquid nitrogen. All samples were stored at – 70°C until ready for use.

### Characterization of an antibody against claudin 7

A rabbit antibody was custom-made against the carboxy-terminal peptide of murine claudin 7, APRSYPKSNSSKEYV, by Zymed Laboratories (San Francisco, CA). This affinity-purified antibody bound only a 23 kDa peptide by western blotting, did not cross-react with claudin 1, its closest congener, in claudin-1-transfected Cos cells or at the apical border of the mammary epithelium where claudin 1 is present at junctional complexes, and stained only epithelia where its mRNA has been demonstrated. Staining was blocked by preincubation of the antibody with the peptide. Cos cells transfected with full-length claudin 7 were stained by this antibody but not by an antibody against claudin 1.

### Protocol for real-time polymerase chain reaction

For real-time reverse transcriptase polymerase chain reaction, RNA was purified with the Qiagen system: 1 μg of DNase-treated RNA was used for each reaction. Triplicate tissue samples were assayed in duplicate with the ABI Prism 7700 sequence detection system, with lung as the positive control. Primer sequences were as follows: murine claudin 7, forward 5' -CGAAGAAGGCCCGAATAGCT-3' (338-337), reverse 5' -GCTACCAAGGCAGCAAGACC-3' (407–388), probe5' -GCCACAATGAAAACAATGCCTCCAGTCA-3' (359–386); murine cytokeratin 19, forward 5' -TTTAAGACCATCGAGGAC-3', reverse 5' -TCATACTGACTTCTCATCTCAC-3'.

### Immunohistochemistry and digital confocal microscopy

Rat anti-mouse ZO1 antibody was obtained from Chemicon International Inc. (Temecula, CA). Tissue sections were cut at 10 μm thickness from frozen blocks with a Damon/IEC division minotome set at – 18 to -20°C. Sections were collected onto Cell-Tak coated coverslips and were further vapor-fixed with paraformaldehyde for 15 min. Sections were never allowed to dry. PBS was added carefully so as to not disrupt the sections. Tissue was permeabilized with 1% Triton X-100 for 15 min, rinsed well with PBS and blocked with sterifiltered 10% normal donkey serum for 20 min. All antibody solutions were microfuged for 20 min before use. The claudin antibody was diluted 1:1000. Primary incubations were for 1 hour at 21–22°C, followed by extensive washes in PBS, generally six times for 5 min each. Secondary antibodies were diluted in accordance with the manufacturer's instructions, in PBS alone. The host species of all secondary antibodies was donkey and all secondary antibodies were cross-adsorbed against mouse serum proteins. Antibodies used were conjugated to fluorescein, CY3 or CY5. Secondary antibodies were combined with 0.6 μg/ml 4,6-diamidino-2-phenylindole, and incubated on the tissue for 1 hour. Coverslips were rinsed briefly and permitted to soak overnight in PBS.

Images were collected with SlideBook software (Intelligent Imaging Innovations, Inc., Denver, CO) on a Nikon Diaphot TMD microscope equipped for fluorescence with a xenon lamp and filter wheels (Sutter Instruments, Novato, CA), fluorescent filters (Chroma, Brattleboro, VT), cooled charge-coupled device camera (Cooke, Tonawanda, NY) and stepper motor (Intelligent Imaging Innovations, Inc., Denver, CO). Multi-fluor images were merged, deconvolved, and renormalized with SlideBook software.

## Results

### Developmental expression of claudin 7 mRNA in the mammary gland

**Figure 1:**
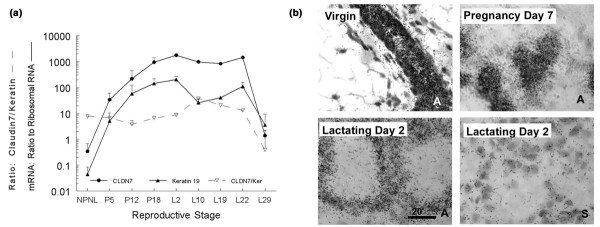
Developmental expression of claudin 7 mRNA in the murine mammary gland. **(a) **Real-time reverse transcriptase polymerase chain reaction measurement of claudin 7 and keratin 19 from total mammary glands of non-pregnant non-lactating (NPNL) mice, mice at 5, 12, and 18 days of pregnancy (P5, P12, and P18), and mice at 2, 10, 19, 22, and 29 days *post partum *(L2, L10, L19, L22, and L29). By L29 the pups have weaned themselves, and mammary gland involution is nearly complete. Dotted line, ratio of claudin 7 RNA to keratin 19 at the same time points. Three mice analyzed at each time point. Error bars represent SEM. Where no bar is visible, the SEM falls within the symbol. Asterisks indicate points that differ significantly from values at L2, *P *< 0.05. **(b) ***In situ *hybridization of claudin 7 probes to sections from virgin, pregnant, and lactating mammary glands. Sections labeled A were hybridized to the anti-sense probe; the lower right-hand section labeled S is the sense control. Scale bar, 20 μm.

Figure 1a shows claudin 7 gene expression as a function of developmental stage in the mouse mammary gland from the virgin animal (non-pregnant non-lactating) through pregnancy (days P5, P12, and P18), lactation (days L2, L10, and L19), and involution (days L22 and L29). Gene expression increased more than 1000-fold between the virgin and early lactating gland, leveling out through lactation and decreasing at late involution (day L29) with the loss of epithelial cells. To determine whether expression of claudin 7 was a function of developmental stage or epithelial cell number, we also examined the expression of keratin 19, found only in luminal epithelial cells [11]. As can be seen, claudin 7 expression parallels that of keratin 19. Further, the ratio of claudin 7 to keratin 19 (Fig. 1a, dotted line) is relatively constant through pregnancy and early lactation, increasing only during later lactation when the expression of keratin 19 mRNA decreases significantly.

At late involution, day L29, claudin 7 expression was lower than that of keratin 19, for reasons that are not clear but probably have to do with the types and characteristics of epithelial cells present during late glandular remodeling. Similar results were obtained from microarray analysis (data not shown). *In situ *hybridization with ^35^S-labeled RNA probes to claudin 7 showed that the mRNA was localized to the epithelium in virgin, pregnant, and lactating animals (Fig. 1b). We conclude that, in the normal mammary gland, claudin 7 is an epithelial cell marker expressed at approximately constant levels through development. The very large changes in expression levels during pregnancy most probably reflect an increase in cell number as the mammary epithelium expands from a system of sparse ducts in the virgin gland, to a dense mass of lactating cells in a gland in which the adipose tissue is largely obliterated.

### Immunolocalization of claudin 7 in the mammary gland

**Figure 2:**
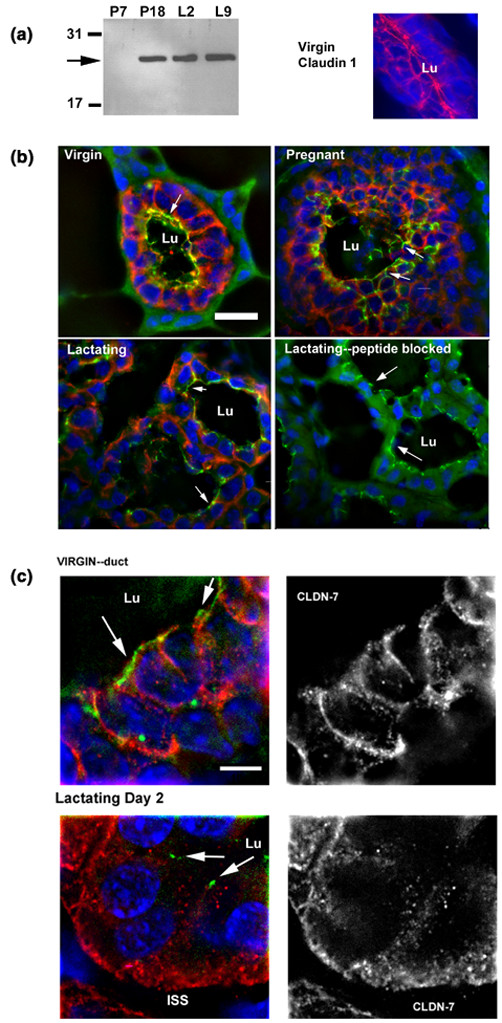
Claudin 7 immunohistochemistry in the mammary gland. **(a) **Western blot on 50 μg of protein from mammary gland at pregnancy days 7 and 18 (P7 and P18) and lactation days 2 and 9 (L2 and L9). Because gene expression is 20-fold lower at P7, as can be seen from Fig. 1a, protein would not be expected to be visible at this exposure. Right-hand side: section of gland from virgin mouse stained with antibody (Zymed) to Claudin 1. **(b) **Lower-power views of epithelial structures from the developmental stages indicated with fluorescein isothiocyanate-labeled ZO-1 (green), Cy3-labeled claudin 7 (red), and 4,6-diamidino-2-phenylindole-labeled nuclei (blue). Lu, lumen; adip, adipocyte. Lower right: anticlaudin antibody blocked with claudin 7 peptide. Scale bar, 30 μm. **(c) **High-power views of claudin 7 localization. The right-hand images show the merged views as in (b). The left-hand black-and-white images show only the claudin 7 stain, demonstrating clearly the punctate nature of the claudin 7 distribution near the basolateral membranes; claudin 7 is excluded from both nuclei and tight junctions. Scale bar, 10 μm. Arrows in all figures point to FITC-stained ZO-1 (green).

We made and characterized an affinity-purified antibody against the cytoplasmic tail of claudin 7 (see Methods) that proved quite satisfactory for both western blots (Fig. 2a) and immunohistochemistry on both frozen and paraffin sections. Given the well-known association of claudins with tight junctions, we were surprised to find that claudin stained the basolateral region of mammary cells in all stages of development (Fig. 2b,c). A ductal structure surrounded by adipose stroma is shown in the virgin; alveoli from the pregnant and lactating glands are also shown (Fig. 2b). Staining was blocked when the antibody was preabsorbed with the claudin 7 peptide against which it was made (Fig. 2b, lower right). As with the *in situ *analysis, stain was observed only in luminal epithelial cells. Although there seems to be some colocalization of claudin 7 with stain for the tight-junction scaffolding protein ZO1 in the lower-power images of Fig. 2b, at higher magnification (Fig. 2c) claudin 7 was entirely localized to the basal and lateral cytoplasmic regions, where it often appeared punctate in nature, particularly when it was not closely apposed to the cell border. Stain was excluded from nuclei (blue) and largely from tight junctions (green), as shown by the minimal overlap between the green and red stain in Fig. 2c. It seems likely that there is basolateral membrane staining in this tissue, but the basal and lateral membranes of the mammary epithelial cells are deeply infolded and membrane localization cannot be assessed at the magnifications possible with the light microscope.

When immunohistochemistry is used in the characterization of claudins, there is always a concern that the antibody cross-reacts with another claudin species. We have found claudins 1, 3, and 8 in the murine mammary epithelium (MC Neville, unpublished data); however, claudin 7 stain shows entirely different patterns from these claudins. As shown in Fig. 2a, claudin 1 is localized at the position of the junctional complexes; claudin 3 is present in epithelium from virgin and pregnant glands, whereas claudin 8 is present only during lactation. These experiments will be described in detail elsewhere.

### Claudin 7 localization in other cell types

**Figure 3:**
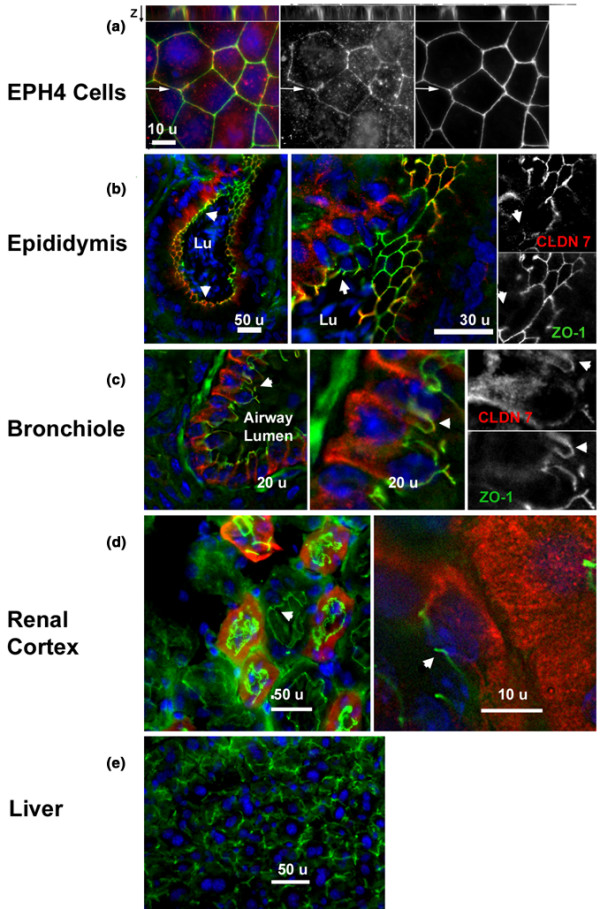
Immunolocalization of claudin 7 (Cy3, red) in cultured mammary epithelial cells, epididymis, lung, kidney, and liver. ZO-1 is stained with fluorescein isothiocyanate to demonstrate location of the tight junctions. **(a) **Eph4 cells. Claudin 7 is found with a patchy distribution at the tight junctions with ZO1. Vesicular stain can also be seen in the cytoplasm. The inset at the top shows the *z *axis across the *xy *view of the section at the level of the arrow. The overlap of ZO1 and claudin indicates colocalization at the level of resolution of the light microscope. **(b) **Mouse epididymis. Claudin 7 stain colocalizes with ZO-1 in most, but not all, tight junctions. Lu, lumen. Small arrowheads in this and subsequent panels denote the apical border of the epithelium. **(c) **Lung. The luminal cells of the bronchiole show heavy staining for claudin 7 in a punctate distribution at the basolateral surface of the cell; however, some claudin 7 colocalizes with ZO-1 (see black-and-white inset). **(d) **Kidney. Some of the tubules of the renal cortex stain as observed by Li and colleagues [7]. Claudin 7 shows a punctate distribution that is heaviest in the basal portion of the cells. **(e) **Liver. Tight junctions at the bile canaliculi are stained with ZO-1. However, no stain for claudin 7 is apparent. The magnification for each figure is indicated by the label (u, μm) above the scale bar.

Sukumar and colleagues [5] reported that claudin 7 was localized to tight junctions of the human mammary carcinoma cell line MCF-7 by using an antibody directed toward the cytoplasmic tail of the human protein, which differs by one amino acid from the mouse protein. We therefore examined claudin 7 staining in the normal mouse mammary cell line EPH4 [12,13]. Figure 3a shows the distribution of claudin and ZO-1 in EPH4 cells. Claudin 7 distinctly colocalizes with ZO-1 with a slightly less compact distribution, as shown by the *zy *image at the top of the figure as well as the *xy *image just below. A scattering of cytoplasmic vesicles containing claudin 7 can also be seen. This finding suggests that claudin 7 is capable of localizing to tight junctions, a conclusion supported by the distribution of claudin 7 in the epididymis (Fig. 3b), where claudin 7 colocalized with ZO-1 at the apical borders of some cells but not others. Punctate cytoplasmic stain for claudin 7 could also be observed in some cells.

Claudin 7 mRNA was reported in lung and kidney, with liver being negative [4]. We therefore examined these tissues and found that claudin 7 was present in cells lining bronchiolar structures of the lung, where its distribution was mainly cytoplasmic as observed previously [8] (Fig. 3c). However, careful examination of a black-and-white image at the highest magnification in the luminal cells of this structure shows some claudin 7 to be colocalized with ZO-1, suggesting that it might also be associated with tight junctions in these cells. There was no stain in the pulmonary alveoli. In the renal cortex we found claudin 7 localized only to certain segments, identified as connecting tubules and cortical collecting ducts by Li and colleagues [7], where stain was distinctly basolateral (Fig. 3d). Because our antibody was different from that used by Li and colleagues, our finding confirms this localization. At high magnification the stain is punctate, as in the mammary and airway epithelia. No specific claudin stain was observed in the liver (Fig. 3e). These findings indicate that claudin 7 is capable of localizing to tight junctions, as in cultured mammary epithelial cells and epididymis; however, in mammary gland, airway, and kidney it is mostly or entirely confined to punctate cytoplasmic structures, often near the basolateral surfaces of the cells and possibly associated with the basolateral membranes. In no tissue was the protein observed in nuclei.

### Claudin 7 localization in mammary tumors

**Figure 4:**
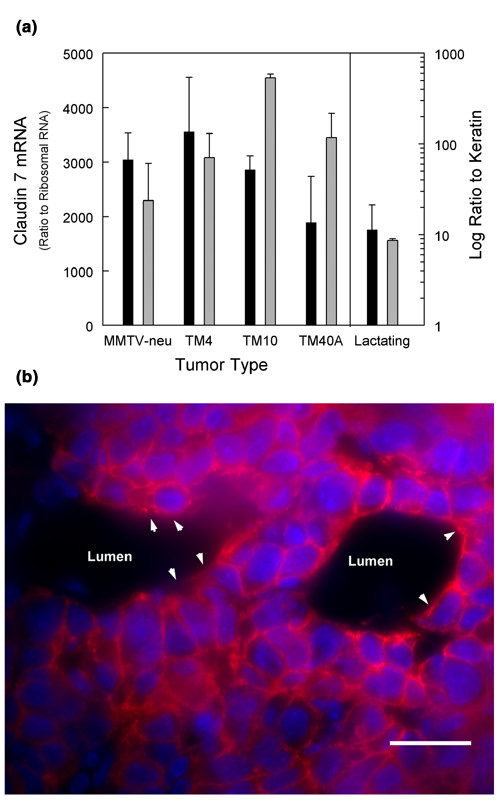
Claudin 7 in mouse mammary tumors. **(a) **Black bars, mRNA levels by real-time reverse transcriptase polymerase chain reaction in the four murine mammary tumors and the lactating gland, chosen for comparison because it, like the tumors, contains a large proportion of epithelial cells. All values are normalized to ribosomal RNA. Gray bars, Ratio of claudin 7 mRNA to keratin 19 mRNA. Error bars represent two SEM. **(b) **Claudin 7 distribution in paraffin section of the transplantable tumor TM4. The stain is localized to the perimembrane cytoplasm in all cells and excluded from nuclei. The arrowheads mark the point in which tight-junction stain would be expected if tight junctions were to form; however, no ZO1 stain was detected in the section. Scale bar, 20 μm.

We examined four types of mammary tumor: tumors arising in the transgenic mouse expressing the Erb2 receptor under the control of the mammary tumor virus promoter [9], and three transplantable tumors obtained from the laboratory of DM [10]. All tumors expressed claudin 7 mRNA at levels no more than twice that in the lactating mammary gland when normalized to ribosomal RNA (Fig. 4a). Although tumors themselves are more closely related to the pregnant gland, we chose the lactating gland for comparison because, like the tumors, it is composed largely of epithelial cells. Interestingly, the ratio of claudin 7 to keratin 19 in the tumors ranged from 4 to 60 times that in the lactating gland, reflecting a lower expression of keratin 19, and possibly a loss of differentiation, in the tumors. In all murine tumors examined here, claudin 7 was localized to the perimembrane region, as illustrated by a section of the TM4 tumor (Fig. 4b). None of these tumors showed ZO1 staining, indicating that they lacked tight junctions, so that the claudin 7 observed here was probably associated with membrane vesicles and possibly with basolateral membranes, as in the normal cells of the mammary gland. Sukumar and colleagues [5] observed a loss of claudin 7 expression in some human tumors, particularly lobular tumors. However, this finding was not true of the mouse mammary tumors examined.

## Discussion

We find claudin 7 to be a constitutive component of the mammary epithelium, where it is localized to the basolateral regions of the cell. The finding that the ratio of its mRNA to that of keratin 19 is relatively constant throughout the developmental cycle suggests that the molecule might be an alternative marker to keratin for the proportion of epithelial cells in the mammary gland. It is not clear whether the more than 1000-fold increase in expression between the virgin gland and early lactation indicates that the number of epithelial cells increases in this proportion, because part of the increase could be a function of an increase in cell size as the cells differentiate. The rapid increase between the virgin gland and pregnancy day 5 is consistent with studies showing a peak of thymidine incorporation between days 2 and 5 of pregnancy, when about 25% of the cells were labeled [14,15]. In both studies, labeling decreased thereafter but remained about 10% almost to the end of pregnancy, consistent with the continued increase in both claudin 7 and keratin mRNA up to day P18.

Our findings are consistent with images of claudin staining in the human mammary gland, where a diffuse diaminobenzidene stain from alkaline phosphatase localization was present throughout the cytoplasm [5]. However, in that study no attempt was made to localize claudin 7 stain with tight-junction components. Basolateral localization of other claudins has been observed. Claudin 1 was localized to the cytoplasm in the epididymis [16], intestine [17], and cornea [18]. Rahner and colleagues [19] observed claudins 3, 4, and 5 to be laterally distributed in various portions of the gastrointestinal track. The finding that claudin 7 is exclusively located in non-tight-junction regions of mammary and renal epithelial cells [7] suggests that claudins might have functions other than the regulation of tight-junction permeability. Our images seem to be the first to show claudin 7 stain at sufficiently high resolution to show punctate cytoplasmic stain in mammary, airway, and renal epithelial cells. Even at this resolution, obtained with digital confocal imaging with a resolution of about 200 nm, it is not possible to discern with certainty whether claudin 7 is inserted into the convoluted basolateral membranes of these cell types. If so, it is possible that the cytoplasmic spots represent vesicles *en route *to and from to the basolateral membranes, where claudin 7 might interact with components of the extracellular matrix. As a precedent, claudin 11, an oligodendrocyte protein, has been shown to interact with α1-integrin and to regulate the proliferation and migration of oligodendrocytes in culture [20]. Other possibilities are that vesicular claudins could regulate tight-junction permeability by sequestering tight-junction regulatory molecules away from tight junctions, or they could be involved in the stabilization of specialized vesicle compartments within the cytoplasm. Matsuda and colleagues, using time-lapse photography, have shown that claudin-containing cytoplasmic vesicles can originate from the tight junctions as the epithelial layer remodels [13]. However, because claudin 7 is never observed in association with tight junctions in mammary epithelial cells, it seems unlikely that these vesicles have an origin in the junctional complex. Our mammary epithelial cell model, EPH4 cells, does possess a complement of cytoplasmic vesicles that could allow live-cell imaging studies to determine the origin and disposition of claudin 7-containing vesicles. It is likely that the function of the vesicles can be better assessed after analysis of the composition of the claudin 7-containing cytoplasmic vesicles.

Claudin 7 expression was inversely correlated with histological grade in a large series of breast tumors [5]. These same authors found, similarly to our observations with EPH4 cells, that claudin 7 colocalized with ZO1 in MCF7 breast cancer cells and could also be observed in cytoplasmic spots. It was present in luminal cells of the human breast, where the diaminobenzidine staining seemed to be localized to basolateral membranes, although it is difficult to draw a firm conclusion in the absence of an apical marker like ZO1. An image of ductal carcinoma *in situ *in that paper shows a distribution of stain remarkably similar to that of the TM10 tumor shown in Fig. 4b. This tumor line has been shown to have a ductal morphology [10].

Thus, we conclude that low-grade breast carcinomas show a cellular distribution of stain similar to that observed in the murine tumors. Interestingly, the murine tumors showed claudin 7 expression at the mRNA level equal to or higher than that of the lactating mammary gland, where the largest proportion of the cells are the luminal epithelial cells that give rise to mammary tumors. TM4, the most tumorigenic of these lines, had the highest claudin 7 expression, although the level was quite variable and not significantly different from the other lines examined. Interestingly, TM4 and the MMTV-*neu *tumor had ratios of claudin 7 to cytokeratin less than one-eighth of that in the slower-growing TM10 line. All of the tumors had claudin 7 to cytokeratin ratios significantly higher than those in the pregnant or lactating mammary gland. These observations suggest that loss of the cytoplasmic architectural stability conferred by keratin might be an early event in tumorigenesis. Together with the data from the Sukumar laboratory [5], we might speculate that loss of claudin 7, as occurs in high-grade tumors, alters cell–matrix interactions, allowing a greater degree of cell mobility and contributing to metastasis.

## Conclusion

Claudin 7 is a constitutive component of mammary epithelium at all stages of development, maintaining a level of gene expression that is approximately proportional to the amount of epithelial tissue in the gland. The protein is not associated with tight junctions but is found in particulate structures in the cytoplasm, generally denser at basolateral borders of the cells. Its distribution is similar in mammary tumors, at least in more well-differentiated ones. Claudin 7 function in the mammary epithelium is currently unknown but might be related to vesicle trafficking or cell-matrix interactions.

## Abbreviations

IHC = immunohistochemistry; PBS = phosphate-buffered saline.

## Competing interests

The author(s) declare that they have no competing interests.

## Authors' contributions

BB performed the real-time polymerase chain reaction and *in situ *experiments and was involved in antibody development and immunocytochemistry. She wrote the initial draft of the manuscript. TR assisted with the immunocytochemistry. SKN provided general molecular knowledge for experimental design and provided valuable input into the manuscript. DM provided the TM series of tumors. MCN conceived the study, performed the high-resolution immunocytochemical analysis and completed the manuscript for submission. All authors read and approved the final manuscript.

## References

[B1] Kubota K, Furuse M, Sasaki H, Sonoda N, Fujita K, Nagafuchi A, Tsukita S (1999). Ca^2+^-independent cell-adhesion activity of claudins, a family of integral membrane proteins localized at tight junctions. Curr Biol.

[B2] Colegio OR, Van Itallie CM, Rahner C, Anderson JM (2003). Claudin extracellular domains determine paracellular charge selectivity and resistance but not tight junction fibril architecture. Am J Physiol Cell Physiol.

[B3] Colegio OR, Van Itallie CM, McCrea HJ, Rahner C, Anderson JM (2002). Claudins create charge-selective channels in the paracellular pathway between epithelial cells. Am J Physiol Cell Physiol.

[B4] Morita M, Furuse M, Fujimoto K, Tsukita S (1999). Claudin multigene family encoding four-transmembrane domain protein components of tight junction strands. Proc Natl Acad Sci USA.

[B5] Kominsky SL, Argani P, Korz D, Evron E, Raman V, Garrett E, Rein A, Sauter G, Kallioniemi OP, Sukumar S (2003). Loss of the tight junction protein claudin-7 correlates with histological grade in both ductal carcinoma in situ and invasive ductal carcinoma of the breast. Oncogene.

[B6] Al Moustafa AE, Alaoui-Jamali MA, Batist G, Hernandez PM, Serruya C, Alpert L, Black MJ, Sladek R, Foulkes WE (2002). Identification of genes associated with head and neck carcinogenesis by cDNA microarray comparison between matched primary normal epithelial and squamous carcinoma cells. Oncogene.

[B7] Li WY, Huey CL, Yu AS (2004). Expression of claudin-7 and -8 along the mouse nephron. Am J Physiol Renal Physiol.

[B8] Coyne CB, Gambling TM, Boucher RC, Carson JL, Johnson LG (2003). Role of claudin interactions in airway tight junctional permeability. Am J Physiol Lung Cell Mol Physiol.

[B9] Guy CT, Webster MA, Schaller M, Parsons TJ, Cardiff RD, Muller WJ (1992). Expression of the *neu *protooncogene in the mammary epithelium of transgenic mice induces metastatic disease. Proc Natl Acad Sci USA.

[B10] Medina D, Kittrell FS, Liu Y-J, Schwartz M (1993). Morphological and functional properties of TM preneoplastic mammary outgrowths. Cancer Res.

[B11] Gudjonsson T, Villadsen R, Nielsen HL, Ronnov-Jesson L, Bissell MJ, Peterson OW (2002). Isolation, immortalization, and characterization of a human breast epithelial cell line with stem cell properties. Genes Dev.

[B12] Reichmann E, Schwarz H, Deiner EM, Leitner I, Eilers M, Berger J, Busslinger M, Beug H (1992). Activation of an inducible c-FosER fusion protein causes loss of epithelial polarity and triggers epithelial-fibroblastoid cell conversion. Cell.

[B13] Matsuda M, Kubo A, Furuse M, Tsukita S (2004). A peculiar internalization of claudins, tight junction-specific adhesion molecules, during the intercellular movement of epithelial cells. J Cell Sci.

[B14] Traurig HH (1967). Cell proliferation in the mammary gland during late pregnancy and lactation. Anat Rec.

[B15] Borst DW, Mahoney WB (1982). Mouse mammary gland DNA synthesis during pregnancy. J Exp Zool.

[B16] Gregory M, DuFresne J, Hermo L, Cyr DG (2001). Claudin-1 is not restricted to tight junctions in the rat epididymis. Endocrinology.

[B17] Walsh SV, Hopkins AM, Chen J, Narumiya S, Parkos CA, Nusrat A (2001). Rho kinase regulates tight junction function and is necessary for tight junction assembly in polarized intestinal epithelia. Gastroenterology.

[B18] Ban Y, Dota A, Cooper LJ, Fullwood NJ, Nakamura T, Tsuzuki M, Mochida C, Kinoshita S (2003). Tight junction-related protein expression and distribution in human corneal epithelium. Exp Eye Res.

[B19] Rahner C, Mitic LL, Anderson JM (2001). Heterogeneity in expression and subcellular localization of claudins 2, 3, 4, and 5 in rat liver, pancreas and gut. Gastroenterology.

[B20] Tiwari-Woodruff SK, Buznikov AG, Vu TQ, Micevych PE, Chen K, Kornblum HI, Bronstein JM (2001). OSP/Claudin-11 forms a complex with a novel member of the tetraspanin super family and α1 integrin and regulates proliferation and migration of oligodendrocytes. J Cell Biol.

